# Heavy Metals and PAHs in Meat, Milk, and Seafood From Augusta Area (Southern Italy): Contamination Levels, Dietary Intake, and Human Exposure Assessment

**DOI:** 10.3389/fpubh.2020.00273

**Published:** 2020-07-07

**Authors:** Calogero Di Bella, Anna Traina, Cristina Giosuè, Davide Carpintieri, Gianluigi Maria Lo Dico, Antonio Bellante, Marianna Del Core, Francesca Falco, Serena Gherardi, Maria Michela Uccello, Vincenzo Ferrantelli

**Affiliations:** ^1^Istituto Zooprofilattico Sperimentale della Sicilia (IZSSi), Palermo, Italy; ^2^National Research Council of Italy-Institute of Anthropic Impacts and Sustainability in Marine Environment (IAS-CNR), Palermo, Italy; ^3^National Research Council of Italy- Institute for Biological Resources and Marine Biotechnology (IRBIM-CNR), Mazara Del Vallo, Italy; ^4^National Research Council of Italy-Institute of Marine Science (ISMAR-CNR), Naples, Italy; ^5^Azienda Sanitaria Provinciale, Siracusa, Italy

**Keywords:** heavy metal, PAH, foodstuff, risk assessment, estimated weekly intake (EWI), target hazard quotient (THQ), cancer risk (CR), margin of exposure (MOE)

## Abstract

Heavy metals and PAHs were measured in animal foodstuffs from Augusta-Melilli-Priolo area in order to evaluate the potential human health risk associated to their consumption. All heavy metals were detected in seafood products while most of them were <LOD in beef, pork and milks samples. Particularly, seafood products registered higher values of total arsenic (As), mercury (Hg) and lead (Pb) than other food categories, while beef and pork showed higher content of zinc (Zn). Cadmium (Cd) and Pb were below the tolerable limits reported by the European Union in foodstuffs (1) while mercury exceed the threshold value in seafood products. Among the PAHs, chrysene (Chr) was detected in all the terrestrial foodstuffs with higher concentrations found in raw milks. Small quantity of benz(a)anthracene (BaA) were also found in this food. The health risk for consumers was assessed for five age categories of consumers calculating the estimated weekly intake (EWI), the target hazard quotient (THQ) and the cancer risk (CR) for each contaminant. Moreover, the margin of exposure (MOE) was estimated for PAHs. The EWI_Hg_ related to seafood products intake exceeded the Provisional Tolerable Weekly Intake (PTWI) recommended by the European Food Safety Authority. The THQ_Hg_ was >1 for baby, children and teenagers, indicating a non-carcinogenic risk for these age categories by seafood ingestion. The CR_As_ overcame 1^*^10^−5^ for almost age categories (except “baby”) and for elderly, by seafood and beef ingestions respectively. Moreover, the MOE for PAHs showed a certain cancer risk for “baby” related to cow milk ingestion.

## Introduction

Industrial activities release into the environment different wastes (gases, particles, sludge, liquid effluent) containing significant amounts of pollutants, such as heavy metals, polycyclic aromatic hydrocarbons (PAHs), polychlorinated dibenzo-p-dioxins (PCDDs) polychlorinated dibenzofurans (PCDFs) and polychlorinated biphenyls (PCBs). Some of them are highly toxic and persistent in the environment, representing a serious threat to human and ecosystem health (2–10).

Heavy metals such as arsenic (As), cadmium (Cd), chromium (Cr), copper (Cu), mercury (Hg) lead (Pb), nickel (Ni), and zinc (Zn) occur in the environment both by natural (i.e., soil erosion and weathering of the earth's crust) and anthropogenic sources (i.e., mining, industrial effluents, urban runoff, sewage discharge, insect or disease control agents applied to crops, and many others). Although some heavy metals (Co, Cu, Cr, Ni, and Zn) play key biological functions in the organisms, they can be potentially toxic if present at higher concentrations. The so called non-essential heavy metals (Cd, Pb, and Hg) are toxic even at very low concentration (11–13). Due to their chemical properties, they can escape cellular control mechanisms, bind to native protein, DNA and nuclear proteins, inhibiting their biological activity resulting in toxicity, oxidative deterioration of biological macromolecules (14–16).

The polycyclic aromatic hydrocarbons (PAHs) are generated from the pyrolysis and incomplete combustion of organic matter (17, 18). They have been classified as genotoxic and possibly/probably carcinogenic to humans (19). Among them the benzo(a)pyrene is the most studied and is classified as human carcinogenic in the Group 1 (19). According to the EU Scientific Committee on Food, the benzo(a)pyrene (BaP) and Σ4PAHs (benzo(a)pyrene + benzo(a)anthracene + chrysene + benzo(b)fluoranthene) can be used as a marker for carcinogenic PAHs in food [(1); subsequent amendments and additions]. The area delimited by the municipalities of Augusta, Melilli and Priolo (SE Italy) is a so-called Site of National Interest (SNI) included in the National Remediation Plan by the Italian Environmental Protection Ministry in 2002 (20). The petrochemical industry of Augusta is considered one of the largest and most complex plants in Europe, located in a bay extending about 20 km along the eastern coastline of Sicily (South Italy). Since 1950, different industrial installations have been allocated, in particular chloralkali plant (1958–2005) and oil refineries, petrochemical and chemical industries, cement plants and electric power stations (20–22), generating an uncontrolled discharge of chemical pollutant in the environment (23). Due to the significant level of environmental degradation, this area is considered a site of high environmental risk, both at Italian (24) and international level (25). Local populations are constantly exposed to different contaminant from several pathways (such as air, water and food). Different studies have shown an alarming increase of congenital malformations, abortions, mortality rates, cancer diseases and nervous system malformations affecting the local populations (25–33). Other authors found high level of Hg and PAHs in sediment from the Augusta Bay (20, 21, 34), exceeding the standard limit reported by national and international sediment quality guidelines (SQGs) (35, 36). Moreover, the contamination effects on marine ecosystems and human population exposed to pollutants through the local fish consumption were studied (20, 21, 23, 34, 37–48). Food ingestion is the most important pathway of contaminant exposure for human and actually seafood and terrestrial animal products are the main route of exposure to heavy metals and persistent organic pollutants to human (49–51). Fish and seafood products are important sources of human diet and have been considered good bioindicators of environmental contamination, because of their ability to accumulate contaminants both by absorption from the environment and food ingestion (52). Similarly, food of terrestrial origin (meat and milk) represents an important source of lipophilic contaminants for human consumers. Different studies showed how these matrices can accumulate significant level of pollutants (such as PAHs, PCBs and heavy metals) into hydrophobic compartments through breathing and ingestion of contaminated water, fodder and soil during the grazing (3, 6, 7, 9, 10, 17, 53–61).

Few data are available on contaminant concentrations in foodstuffs from the area of Augusta-Melilli-Priolo, as well as studies aimed to estimate the potential public health risks for local consumers. Therefore, in this study we evaluate heavy metals and PAHs concentrations in seafood products, meats and milks samples collected from the SNI area, in order to assess the human health risk for resident population due to the consumption of local animal foodstuffs.

## Materials and Methods

### Samples Collection

A total of 96 samples of different fish species (*Pagellus bagaraveo, Mullus barbatus, Trigla lucerna, Pagellus erythrinus, Sphyrena sphyrena, Diplodus annularis, Diplodus sargus, Pagellus acarne)* molluscs (*Sepia officinalis*), and crustaceans (*Parapaeneus kerathurus*) were collected during 2018 from local markets and during capture. After collection, they were packed, frozen at −20°C and stored until delivery to the laboratories for further analysis. Specimens with similar size were pooled as single sample and dissected by means of stainless steel scissors in order to avoid contamination (Table 1). The edible part of pooled samples was removed, homogenized, and freeze-dried for chemical analysis. Chemical analyses seafood products were conducted at the CNR's laboratories.

**Table 1:** Species, number of individuals and pool of fish, mollusc, and crustaceans analyzed for the category “Seafood products.”

**Specie**	**Common name**	**Total individuals**	**Pool**
**Fish**			
*Pagellus erythrinus*	Common pandora	28	5
*Pagellus acarne*	Axillary seabream	8	2
*Pagellus bogaraveo*	Blackspot seabream	1	1
*Mullus barbatus*	Red mullet	6	2
*Diplodus annularis*	Annular seabream	2	1
*Diplodus sargus*	White seabream	2	1
*Trigla lucerna*	Tub gurnard	2	1
*Sphyraena sphyraena*	European barracuda	1	1
**Molluscs**			
*Sepia officinalis*	Common cuttlefish	7	6
**Crustaceans**			
*Penaeus kerathurus*	Caramote prawn	39	4

**Figure 1:**
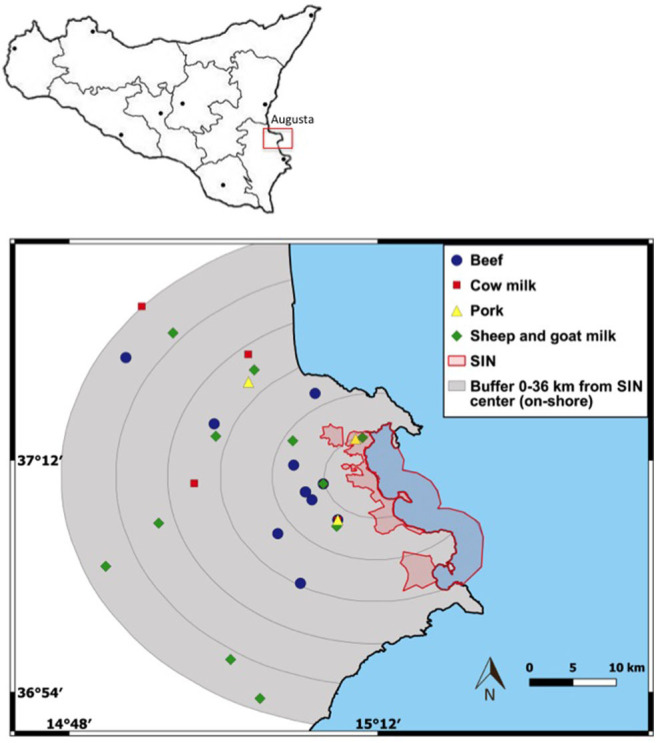
Mapping of terrestrial foodstuff samples and the SNI area.

From May to August 2018, a total of 30 samples of bulk milks and meats (5 bovine milk, 11 sheep and goat milk, 11 beef and 3 pork meats samples) were collected from 26 different farms located in the SNI area. Particularly, the milks were taken directly in the farms, while the meats in the slaughterhouses. Different criteria were adopted to select the farms from the Italian veterinary data bank after a preliminary study on the livestock productions realized in this area. The following conditions were considered: adoption of extensive management systems, number of animals/farm > 5 (farms with higher number of animals were preferred) and geographical characteristics of their location (morphology and soil use), considering semi-circular buffers of 6 km (from 0 to 36 km from the center of the SNI - Figure 1). After collection, samples were immediately frozen at −20°C (Thermo scientific: Thermo GPS Series) and storedand analyzed [in accordance with (62)] at the laboratories of Istituto Zooprofilattico Sperimentale della Sicilia (IZSSi).

### Chemical Analysis

#### Seafood Samples

About 0.25 gr of the dried and powdered tissue of seafood products were digested using concentrated nitric acid in microwave (CEM EXPLORER SPD). The digested samples were diluted to 50 ml with deionized water and contents of As, Cd, Cr, Ni, Pb, and Zn were determined by ICP-MS (Icap Q- Thermo-Icap). The accuracy of the method was validated by the analysis of Certified Reference Materials for lobster hepatopancreas (TORT-2, National Research Council Canada). Standards were analyzed every 10 samples and all runs were carried out in triplicated. The accuracy was between 0.4 and 15%. The analytical precision, based on triplicated runs (RSD%, *n* = 3) was <10% and the reproducibility was better than 7% (LODs μg/g: As 0.001; Cd 0.001; Cr 0.002; Ni 0.004; Pb 0.004; Zn 0.06). The concentration of Hg was determined by a direct mercury analyzer atomic absorption spectrophotometer (milestone-DMA-80®), according to analytical procedures reported in EPA 7473. About 0.05 g of dried tissue was loaded in nickel boats and transferred to the DMA-80® system. Acid-cleaned laboratory materials were used in order to minimize contamination risks, during sample preparation and analyses procedures. The Certified Reference Materials-TORT-2 was used to assess analytical accuracy (estimated to be 3%) and precision (routinely better than 4%; RSD%, *n* = 3). Finally, about 20% of the total number of samples were duplicated to estimate reproducibility (which resulted in better than 7%) (LOD 0.2 μg/kg).

For PAHs determination, 1 g of freeze-dried of muscle spiked with a 45 ng of deuterated standard of PAHs, was extracted by Accelerated Solvent Extraction (ASE 200, DIONEX, Thermo Scientific) using a hexane/acetone (80:20 v/v) mixture. The extract was subjected to a saponification reaction by adding sodium hydroxide 6M, concentrated and re-dissolved with 1 ml of cyclohexane. Subsequently it was purified by SPE cartridges containing 6 g silica and cyclohexane:acetone (70:30) mixture. The final extracts were analyzed by Gas Chromatography (GC-MS ISQ; Thermo Finningan) with Mass Spectrometric detection in Selective Ion Monitoring (SIM) mode. Laboratory quality control procedures included analyses of blanks, reference material and spiked samples. The reference material used for quality control was SRM 2974a NIST (recoveries for each analyte ranged between 54 and 111%). The accuracy estimated on multiple analysis of the reference material was estimated at more than ± 10% for each single analyte. The precision estimated on triplicate samples was >90% and the reproducibility was better than 10%. The limit of detection of the method was estimated as 0.8 ng/g for each PAH. All results were converted from dried to wet-weight (μg g^−1^) applying a conversion factor previously calculated using the following formula: C_w_ = C_d_ x (100-%H_2_O/100) were C_d_ and C_w_ are the concentration expressed relatively to dry and wet mass, respectively. %H_2_O is the percentage of humidity in wet tissues calculated after the freeze-drying process [ranging around 80% for almost species—(63–65)].

#### Milk and Meat Sample

Heavy metals (As, Cd, Cr, Ni, Pb, and Zn) in milk and meat samples were determined by ICP-MS (7700x series, Agilent Technologies, Santa Monica CA, USA) following the method reported by Lo Dico et al. (66), validated in according to Lo Dico et al. (67). About 1 g of sample was transferred into previously decontaminated PTFE vessels with 3 ml of 65% ultrapure nitric acid (V/V) and 5 ml of deionized water, and subsequently mineralized by microwave digestion (Multiwave 3000 Anton-Paar, Graz, Austria). The extracts were diluted to 50 ml, filtered and analyzed by ICP-MS. A pool of digested samples was used for the test and a calibration curve was made to evaluate the linearity with 8 standard points (BlankCal - 0.01 – 0.05 – 0.1 – 0.5 – 1 – 5 – 10 – 50 μg/l).

Mercury (Hg) concentrations were measured using a direct mercury analyser atomic absorption spectrophotometer (DMA-80®). Aliquots of 0.09 ± 0.01 g (w/w) of each thawed sample was homogenized and added into nickel boats and introduced in the DMA-80® direct analyser (Milestone, Bergamo, Italy). The amount of mercury present was detected and quantified according to calibration curves of 5 concentration points (0.050–2 mg/Kg). Standards for the instrument calibration were prepared on the basis of mono-element certified reference solution ICP standard (VWR, Milan, Italy). The limits of detection and quantification (LOD and LOQ), the repeatability and recovery of the method were calculated as described by Lo Dico et al. (66) (Table 2).

**Table 2:** Validation parameters for terrestrial foodstuffs.

**Metals**	**LOD**		**LOQ**		**Repeatability**		**Recovery %**	
	**Meats (mg/kg)**	**Milk (mg/kg)**	**Meats (mg/kg)**	**Milk (mg/kg)**	**Meats (mg/kg)**	**Milk (mg/kg)**	**Meats**	**Milk**
Arsenic (As)	0.003	0.003	0.004	0.003	0.0016	0.0015	100.3	99.7
Cadmium (Cd)	0.003	0.001	0.004	0.001	0.0002	0.0013	102.0	100.3
Chromium (Cr)	0.070	0.070	0.090	0.090	0.0200	0.0200	106.3	106.3
Mercury (Hg)	0.041	0.041	0.050	0.050	0.0210	0.0210	103	103
Nickel (Ni)	0.050	0.050	0.060	0.060	0.1330	0.1330	92.0	92.0
Lead (Pb)	0.003	0.003	0.004	0.003	0.0016	0.0013	100.3	100.3
Zinc (Zn)	0.067	0.067	0.079	0.079	0.6820	0.0900	94.0	94.0
PAHs	200.0	200.0	500.0	500.0	60.00	60.000	75.0	75.0

Extraction of PAHs, was carried out as follow: an aliquot of 5.00 ± 0.10 g of homogenized sample was taken and weighed in a 50 ml disposable tube by an analytical balance. Into the same tube, 50 μl of Mix SI PAHs (BaA-d12 and BaP-d12) were added to 100 μg/l (prepared at the time of use) of a buffer salts mixture used for the extraction phase (SUPELCO Acetate Extraction Tube cat. 55234-U or equivalent). The sample was centrifuged for 5 min at a speed of 4,000 rpm. The supernatant was transferred into a 15 ml centrifuge tube containing the purification step (SUPELCO QuE Z-Sep/C18 Cleanup Tube cat. 55401-U or equivalent) for purification and subsequently stirred for a minute and centrifuged for 5 min at a speed of 4,000 rpm. After removing the supernatant, it was transferred to a 10 ml glass tube and evaporation was made in a nitrogen stream at a temperature of 30–35°C. The purified formed residue was then recovered with 500 μl of Chrysene-d12 syringe standard at 10 μg/l, prepared at the time of use and transferred into 2 ml amber vials with conical insert for GC autosampler. An automatic GC/MSMS analyzer (Thermo Scientific TSQ Quantum XLS Triple Quadrupole GC/MSMS) was used to perform the detection. An aliquot of 2 μl and a control standard of 10 μg/l were inserted (calibration standard: 1–2–5–10–20–50–100 μg/l) in the instrument. For the calculation of the concentration of analyte in the matrix a conversion factor equal to 0.5/Sample weight was calculated. The limits of detection and quantification (LOD and LOQ), the repeatability and recovery of the method were calculated as described by Lo Dico et al. (66) and reported in Table 2. The values obtained were the same for each PAH (benzo(a)pyrene, benz(a)anthracene, benzo(b)fluoranthene, and chrysene).

The quality of analytical procedures is in according to (62) and the ISO ENI 17025: 2018. The validity of the method was performed by proficiency test samples (Zscore <2) (Fapas®: Food Chemistry Proficency Test Report 07318, 2018, for Heavy Metals in milk powder; Fapas®: Food Chemistry Proficency Test Report 0677, 2018, for PAHs in smoked fish). In addition, Certified Reference Material DORM-4 (fish protein) was analyzed for analytical batch (Table 3). The repeatability limit was lower than the value obtained in the validation process through the analysis of double samples (C_1_-C_2_ < r; where C are the concentrations of the samples analyzed in duplicate and r is the limit of repeatability at that level). Finally, the accuracy, precision and reproducibility were lower than the limits calculated in the validation process (66–68) (Table 3).

**Table 3:** Quality parameters for chemical analysis of terrestrial foodstuffs.

**Heavy metals**	**DORM-4 certified values (μg/g)**	**DORM-4 obtained values (μg/g)**	**Proficiency test 07318 certified values for milk (μg/kg)**	**Proficiency test 07318 obtained values for milk (μg/kg)**	**Z-score (μg/kg)**
Arsenic (As)	6.87 ± 0.44	6.22 ± 0.51	76.4	65.00	−0.7
Cadmium (Cd)	0.299 ± 0.018	0.301 ± 0.020	24.9	21.00	−0.7
Lead (Pb)	0.404 ± 0.062	0.411 ± 0.048	70.4	70.00	0.0
Mercury (Hg)	–	–	34.9	42.00	0.9
**PAHs**			**Proficiency test 0677 certified values for smoked fish (μg/kg)**	**Proficiency test 0677 obtained values for smoked fish (μg/kg)**	**Z-score (μg/kg)**
benzo(a)pyrene			3.20	2.36	−1.2
benzo(a)anthracene			11.5	10.37	−0.5
chrysene			13.6	17.96	1.5
benzo(b)fluoranthene			5.29	5.26	0.0

### Statistical Analysis

Results of heavy metals in terrestrial foodstuffs were integrated with those of the National Residual Plan (2012–2015) for the municipalities of Siracusa, in order to obtain a more representative dataset for the SNI area. Therefore, a statistical analysis was performed by SAS (9.1 version; non-parametric Mann-Whytney U-test by NPAR1WAY, *p* ≤ 0.05) to compare the heavy metal concentrations of terrestrial foodstuffs of the SNI area with those detected in the same matrices from other districts of Sicily(National Residual Plan 2012–2015-IZSSi database). Values < LOD were considered as ½ LOD (69).

## Estimation of Potential Public Health Risks

A human health risk assessment was conducted according to the United States Environmental Protection Agency methods (70), considering the ingestion rates of five different age-categories of consumers (71), namely children 0–2 years (11.3 kg), children 3–9 years (26.1 kg), adolescent 10–17 years (52.6 kg), adult 18–64 years (69.7 kg), seniors 65–97 years (70.1 kg) (Table 4).

**Table 4:** Ingestion rates of animal foodstuffs, considering five age-categories of Sicilian consumers (71).

**Population group (men and female) SICILY (Italy)**	**BW**	**Beef**	**Pork**	**Milk**	Seafood^*^
**Age-categories**	**(kg)** Mean	**(g/kg BW/die)** **Mean ± SD**			
Baby (0–2 years old)	11.3	3.14 ± 5.18	0.13 ± 0.28	49.72 ± 75.39	1.82 ± 2.49
Children (3–9 years old)	26.1	1.04 ± 1.01	0.62 ± 0.95	9.29 ± 7.23	1.70 ± 1.96
Teenagers (10–17 years old)	52.6	0.81 ± 0.78	0.32 ± 0.58	3.36 ± 2.20	1.28 ± 1.41
Adult (18–64 years old)	69.7	0.50 ± 0.54	0.24 ± 0.40	1.46 ± 1.46	0.71 ± 0.80
Elderly (65–97 years old)	70.1	0.54 ± 0.47	0.17 ± 0.37	1.82 ± 1.62	0.54 ± 0.65

* *Unprocessed and frozen*.

The sheep and goat milks are not consumed unprocessed but used for cheese making. Heavy metals and PAH concentrations were detected on fresh milk. It was not possible to estimate their concentration in processed products due to the lack of the conversion factors from milk to cheese. Therefore, sheep and goat milks were excluded in the estimation of potential human health risk. Concerning the risk exposure due to the seafood consumption, we considered the average values of each pollutant, independently from the specie (as indicative of “Seafood products”) in according to the foodstuffs categories reported by INRAN (71).

The human exposure of heavy metals and PAHs due to animal products ingestion was assessed calculating the Estimate Weekly Intake (EWI), the Target Hazard Quotient (THQ) and the Lifetime Cancer Risk (CR). According to previous studies, we assumed that the ingestion dose is equal to the adsorbed contaminant dose and that cooking has no effect on contaminant concentrations (72). The EWI, the THQ and the CR for each contaminant (inorganic and organic) were calculated on mean concentrations, considering ½ LOD where data were <LOD (69). The EWI for each metal was compared with the Provisional Tolerable Weekly Intake (PTWI) recommended by the European Food Safety Authority and WHO (Cd = 25 μg/kg bw; Cr = 700 μg/kg bw; Ni = 35 μg/kg bw; Pb = 25 μg/kg bw; Zn = 7,000 μg/kg bw).

Concerning the Hg exposure, we considered the PTWI for methylmercury (MeHg), the most toxic organic form of Hg (73–75) present in fish as more than 70% of total Hg (76–78).

The inorganic form of As (iAs) is considered more toxic than organic arsenic compounds and predominant in terrestrial animal foodstuffs, together with single methylated arsenic species. In this survey, iAs contents in seafood products was estimated applying 2% of the total As (79–86). Actually, the organic As species are commonly present in seafood (i.e., arsenobetaine and different arsenosugars) and considered less dangerous for human health (87, 88). Otherwise, the iAs in terrestrial foodstuffs was calculated as 70% of the total As according to EFSA (89). Therefore, we estimated the iAs human exposure comparing the dietary intake with the lower limit on the benchmark dose for a 0.5% (BMDL0.5) (range: 2–7 μg/kg bw per day) calculated by JECFA (90) for cancer incidence.

### Estimated Weekly Intake

The metals human exposure was assessed according to the following equation:

(1) $$\begin{array}{r} \text{EWI }=\;\left(\text{Cm x I}{\text{R}}_{\text{w}}\right)/\text{BW} \end{array}$$

where Cm represents the average concentrations of contaminant for each category of considered food (μg g^−1^); IR_w_ is the weekly ingestion rate (g week^−1^) derived from the INRAN database (2010) for seafood products, cow milk and meats in Sicily; BW is the body weight (kg) reported by INRAN database (2010) for 5 different age-category of consumers.

The total EWI for each metal derived from meat (beef and pork), cow milk and seafood products ingestion was calculated as follow:

(2) $$\begin{array}{l} {\text{Total EWI}}_{\text{c}}={\text{EWI}}_{\left(\text{c}-\text{beef}\right)}+{\text{EWI}}_{\left(\text{c}-\text{pork}\right)}+{\text{EWI}}_{\left(\text{c}-\text{milk}\right)}\\ \;+{\text{EWI}}_{\left(\text{c}-\text{seafood}\right)} \end{array}$$

To assess the risk due to PAHs exposure, the total BaP equivalent concentration (BEC) was estimated in each foodstuff as follow:

(3) $$\begin{array}{r} \text{BEC}=\overset{n}{\underset{i=1}{\sum }}c_{i}\;x\;TEF_{i} \end{array}$$

Where *c*_*i*_ is the concentration of PAH congener *i* in the foodstuff and TEF is the toxic equivalency factor used to quantify the carcinogenicity of BaA (0.1), BbF (0.1), and Chr (0.01) respect to BaP (91).

The BEC was used to estimate the EWI of all congeners (∑4PAH):

(4) $$\begin{array}{r} \text{EWI}=\left(\text{BE}{\text{C}}^{*}{\text{IR}}_{\text{w}}\right)/\text{BW} \end{array}$$

Similarly to metals, the total EWI was calculated according to the Equation 2.

### Non-carcinogenic Health Hazard

The risk of non-carcinogenic effects was estimated using the Target Hazard Quotient (THQ), that is the ratio between the exposure and the reference dose (RfD). THQ was calculated according to the USEPA (92) method using the following equation:

(5) $$\begin{array}{r} \text{THQ}=\left[\left(\text{EF x ED x FIR x C}/\text{RfD x BW x AT}\right)\right]\text{ x }10^{-3} \end{array}$$

where EF is the exposure frequency (365 days year^−1^ for people who eat each categories of food every day); ED is the exposure duration (years); FIR is the food ingestion rate for each categories of food (g day^−1^) (71); C is the metal concentration in foodstuff (μg g^−1^); RfD is the oral reference dose in μg g^−1^day^−1^: As = 3.0 × 10^−4^, Cd = 1.0 × 10^−3^, Cr = 3 × 10^−3^; Hg = 1.0 × 10^−4^, Ni = 2 × 10^−2^; Pb = 4.0 × 10^−3^; Zn = 3.0 × 10^−1;^ BaP = 0.0003 (92); AT is the average time for non-carcinogens (it is equal to 365 days year^−1^ x ED). The ED used in this study were 1 year (children 0–2 years), 6 years (children 3–9 years), 14 years (adolescent 10–17 years), 41 years (adult 18–64 years), 81 years (seniors 65–97 years).

The THQ for ∑4PAH was calculated by replacing the concentration of metal “C” with the BEC value.

A THQ value <1 indicates negligible non-carcinogenic risks for consumers (72). Higher THQ values indicate significant risks for long-term non-carcinogenic effects (93, 94). Additionally, the total target hazard quotient (TTHQ) for each contaminant (Equation 6) end for all considered toxicants (Equation 7) were also calculated in order to evaluate the non-carcinogenic risk for human resulting from consumption of different foodstuffs:

(6) $$\begin{array}{l} \text{TTH}{\text{Q}}_{\text{c}}=\text{TH}{\text{Q}}_{\left(\text{c}-\text{beef}\right)}+{\text{THQ}}_{\left(\text{c}-\text{pork}\right)}+{\text{THQ}}_{\left(\text{c}-\text{milk}\right)}\\ \;+{\text{THQ}}_{\left(\text{c}-\text{seafood}\right)} \end{array}$$

(7) $$\begin{array}{l} {\text{TTHQ}}_{\text{i}}=\sum \text{THQi} \end{array}$$

### Carcinogenic Risk Assessment of Metals and PAHs

The cancer risk (CR) associated with consumption of selected foodstuff was assessed following the equation:

(8) $$\begin{array}{r} \text{CR}=\left[\left(\text{EF x ED x FIR x C x }{\text{C}}_{\text{S}}\text{F}\right)/\left(\text{BW x AT}\right)\right]\text{ x }10^{-3} \end{array}$$

Were CsF is the cancer slope factor derived by response-dose curve for toxicant ingestion: As = 1.5 kg-day/mg; Cd = 6.3 kg- day/mg; BaP = 1 kg-day/mg (70); Cr = 5 × 10^−1^ kg-day/mg; Pb = 8.5 × 10^−3^ kg-day/mg) (95, 96); Ni = 0.91 kg-day/mg (95).

The CR for ∑4PAH was calculated by replacing the concentration of metal “C” with the BEC value.

Usually, the CR between 10^−6^ (risk of developing cancer over a human lifetime is 1 in 1,000,000) and 10^−4^ (risk of developing cancer over a human lifetime is 1 in 10,000) indicate a low health risk for carcinogens, while a value more than 10^−4^ involves a serious potential health risk (97, 98). In this study, we consider 10^−5^ as cancer benchmark.

Furthermore, the margin-of-exposure approach (MOE) was used according to the EFSA Panel on Contaminants in the Food Chain (CONTAM Panel) (99). The MOE is a useful tool for health risk characterization for a given population exposed to genotoxic and carcinogenic substances as PAHs (99) and is defined as:

(9) $$\begin{array}{r} \text{MOE }=\text{ BMD}{\text{L}}_{10}/\text{EDI} \end{array}$$

where BMDL_10_ represents the lower bound of the 95% confidence interval on the benchmark dose corresponding to a 10% to more incidence in experimental animals (BaP = 0.1 mg /kg BW/day) (99), while EDI stands for the chronic daily dietary PAHs exposure (mg/kg BW/day). Similarly to the other risks indices, the total MOE for Σ4PAH was calculated considering the BEC value. MOEs <10,000 represent a potential concern for human health (99).

## Results

### Heavy Metals and PAHs Content

Heavy metal shows wide range of concentrations among the analyzed samples (Table 5). In general, all heavy metals were detected in seafood while most of them were < LOD in beef, pork and milks (Table 5). Particularly, higher values of total As (mean 7.06 μg/g), Hg (mean 0.99 μg/g) and Pb (mean 0.09 μg/g) were found in seafood products respect to the other categories of foodstuff, while beef and pork showed higher content of Zn (mean 48.94 μg/g and 44.91 μg/g, respectively).

**Table 5:** Mean, standard deviation, and range values of heavy metals and PAHs in the animal foodstuffs.

**Food**	**Heavy metals Means ± SD (range) μg/g**							**PAHs Means ± SD (range) ng/g**			
	**As**	**Cd**	**Cr**	**Hg**	**Ni**	**Pb**	**Zn**	**BaA**	**BaP**	**BbF**	**Chr**
**Beef**	0.012 ± 0.008 (0.002–0.031)	< LOD	< LOD	< LOD	< LOD	0.019 ± 0.027 (0.002–0.098)	48.94 ± 11.94 (32.11–70.15)	< LOD	< LOD	< LOD	0.34 ± 0.65 (0.10–2.26)
**Pork**	0.015 ± 0.012 (0.002–0.022)	< LOD	< LOD	< LOD	< LOD	0.024 ± 0.034 (0.002–0.064)	44.91 ± 13.43 (34.17–59.96)	< LOD	< LOD	< LOD	< LOD
**Cow milk**	< LOD	< LOD	< LOD	< LOD	< LOD	0.002 ± 0.001 (0.001–0.004)	2.92 ± 0.96 (2.06–4.44)	0.30 ± 0.46 (0.10–1.12)	< LOD	< LOD	12.56 ± 19.17 (0.10–43.96)
**Sheep and goat milk**	< LOD	< LOD	< LOD	< LOD	< LOD	0.002 ± 0.002 (0.001–0.007)	2.77 ± 1.18 (1.69–5.18)	0.13 ± 0.09 (0.10–0.39)	< LOD	< LOD	9.25 ± 28.49 (0.10–95.12)
**Fish**											
*Pagellus erythrinus*	3.62 ± 0.98 (2.73–5.02)	0.001 ± 0.0002 (0.0003–0.001)	0.019 ± 0.007 (0.01–0.03)	1.13 ± 0.22 (0.94–1.45)	0.002 ± 0.001 (0.0003–0.003)	0.007 ± 0.003 (0.003–0.01)	3.40 ± 0.76 (2.84–4.66)	< LOD	< LOD	< LOD	< LOD
*Pagellus acarne*	4.72 ± 0.52 (4.35–5.09)	< LOD	0.02 ± 0.02 (0.005–0.04)	0.59 ± 0.11 (0.52–0.67)	0.01 ± 0.006 (0.009–0.018)	0.57 ± 0.079 (0.52–0.63)	2.46 ± 0.26 (2.28–2.64)	< LOD	< LOD	< LOD	< LOD
*Pagellus bogaraveo*	2.47	0.0005	0.01	0.06	0.016	0.02	3.72	< LOD	< LOD	< LOD	< LOD
*Mullus barbatus*	9.94 ± 2.65 (8.1–11.8)	0.001 ± 0.00002 0.001	0.02 ± 0.01 (0.02–0.03)	1.91 ± 0.28 (1.71–2.11)	0.003 ± 0.001 (0.002–0.004)	0.03 ± 0.001 0.03	4.14 ± 1.55 (3.05–5.24)	< LOD	< LOD	< LOD	< LOD
*Diplodus annularis*	4.52	0.001	0.04	3.51	0.001	0.01	6.31	< LOD	< LOD	< LOD	< LOD
*Diplodus sargus*	7.42	0.001	0.01	0.48	0.003	0.17	3.00	< LOD	< LOD	< LOD	< LOD
*Trigla lucerna*	4.41	0.0004	0.02	0.66	0.002	0.01	4.22	< LOD	< LOD	< LOD	< LOD
*Sphyraena sphyraena*	1.37	0.006	0.01	0.78	0.003	0.001	4.40	< LOD	< LOD	< LOD	< LOD
**Molluscs**											
*Sepia officinalis*	22.68 ± 14.88 (8.73–44.60)	0.003 ± 0.002 (0.001–0.006)	0.01 ± 0.01 (0.002–0.02)	0.21 ± 0.08 (0.07–0.78)	0.05 ± 0.03 (0.003–0.09)	0.09 ± 0.11 (0.001–0.29)	13.94 ± 1.88 (4.40–16.22)	< LOD	< LOD	< LOD	< LOD
**Crustaceans**								< LOD	< LOD	< LOD	< LOD
*Penaeus kerathurus*	9.45 ± 2.67 (7.90–13.45)	0.003 ± 0.00 (0.002–0.003)	0.02 ± 0.01 (0.02–0.03)	0.59 ± 0.02 (0.56–0.62)	0.01 ± 0.00 (0.004–0.01)	0.01 ± 0.01 (0.01–0–02)	18.05 ± 4.00 (15.67–24.03)	< LOD	< LOD	< LOD	< LOD
**Total seafood products**	7.06 ± 6.17 (1.37–44.60)	0.002 ± 0.002 (0.0003–0.006)	0.019 ± 0.011 (0.002–0.044)	0.99 ± 1.02 (0.057–3.51)	0.010 ± 0.014 (0.0004–0.085)	0.09 ± 0.18 (0.001–0.63)	6.36 ± 5.27 (2.28–24.03)	< LOD	< LOD	< LOD	< LOD

Focusing on individual marine organisms, Hg concentrations exceed the threshold limits imposed by European Community (1) for seafood in almost the fish species (*P. erythrinus:* 1.13 μg/g; *D. annularis:* 3.51 μg/g; *M. barbatus*: 1.91 μg/g; *T. lucerna:* 0.66 μg/g and *S. sphyraena*: 0.78 μg/g) and in the crustacean *P. kerathurus* (0.59 μg/g), while Pb content was above the threshold limit for fish [0.3 μg/g; (1)] only in the specie *P. acarne* (0.57 μg/g). Cd concentrations remain within the normative limits in all species (Table 5).

The highest values of Pb were found in meats (beef: 0.098 μg/g; pork: 0.064 μg/g) sampled in the farm located at about 6 km from the SNI.

As regard PAHs concentrations, the BaA was detected in cow (mean 0.30 ng/g), sheep and goat milks (mean 0.13 ng/g), while Chr was found in beef (men 0.34 ng/g), cow (mean 12.56 ng/g) and in sheep and goat milks (9.25 ng/g). The BaP and the BbF were <LOD in all foodstuff.

In particular, highest values of chrysene (43.96 ng/g and 95.12 ng/g in cow and sheep and goat milks, respectively) were found in milks collected in the farms located very closed to the SNI.

### Dietary Exposure and Health Risk

The total EWI for all metals were below the PTWI except for Hg, mainly related to seafood ingestion (Table 6). Similarly, values of TTHQ_Hg_ > 1 were obtained in all age classes of consumers primarily due to the intake of milk and seafood products. In particular, THQ_Hg_ > 1 were recorded in 2 age classes due to milk consumption (10.19 and 1.90 for baby and children, respectively) and in 3 age classes due to seafood consumption (2.38, 2.23, and 1.68 for baby, children and teenagers, respectively). Moreover, As and Zn showed a TTHQ > 1 in the age class “baby.”

**Table 6:** Estimated weekly intake (EWI; μg/kg b.w.), Target hazard quotient (THQ), Total Target hazard quotient (TTHQ) and Target carcinogenic risk (CR) for each analyzed metals in the five age-categories considering an exposure time of 365 days year^−1^.

**Food**	**EWI (μg/kg BW)**								**THQ**							**CR**				
		**As**	**Cd**	**Cr**	**Hg**	**Ni**	**Pb**	**Zn**	**As**	**Cd**	**Cr**	**Hg**	**Ni**	**Pb**	**Zn**	**As**	**Cd**	**Cr**	**Ni**	**Pb**
**Beef**	Baby (0–2 yr)	0.35	0.03	0.77	0.45	0.55	2.16	262.40	0.16	0.00	0.04	0.64	0.00	0.02	0.51	1.1E-06	4.2E-07	7.9E-07	1.0E-06	7.2E-09
	Children (3–9 yr)	0.11	0.01	0.25	0.15	0.18	0.71	86.91	0.05	0.00	0.01	0.21	0.00	0.01	0.17	2.1E-06	8.4E-07	1.6E-06	2.0E-06	1.4E-08
	Teenagers (10–17 yr)	0.09	0.01	0.20	0.12	0.14	0.56	67.69	0.04	0.00	0.01	0.17	0.00	0.01	0.13	3.8E-06	1.5E-06	2.8E-06	3.7E-06	2.6E-08
	Adult (18–64 yr)	0.06	0.01	0.12	0.07	0.09	0.34	41.78	0.03	0.00	0.01	0.10	0.00	0.00	0.08	6.9E-06	2.8E-06	5.1E-06	6.7E-06	4.7E-08
	Elderly (65–97 yr)	0.06	0.01	0.13	0.08	0.09	0.37	45.13	0.03	0.00	0.01	0.11	0.00	0.00	0.09	**1.5E-05**	5.9E-06	**1.1E-05**	**1.4E-05**	1.0E-07
**Pork**	Baby (0–2 yr)	0.01	0.00	0.03	0.02	0.02	0.06	40.87	0.00	0.00	0.00	0.03	0.00	0.00	0.02	3.0E-08	1.8E-08	3.3E-08	4.2E-08	3.8E-10
	Children (3–9 yr)	0.05	0.01	0.15	0.09	0.11	0.28	194.90	0.02	0.00	0.01	0.13	0.00	0.00	0.09	8.4E-07	5.0E-07	9.3E-07	1.2E-06	1.1E-08
	Teenagers (10–17 yr)	0.02	0.00	0.08	0.05	0.06	0.14	100.59	0.01	0.00	0.00	0.07	0.00	0.00	0.05	1.0E-06	6.1E-07	1.1E-06	1.5E-06	1.3E-08
	Adult (18–64 yr)	0.02	0.00	0.06	0.03	0.04	0.11	75.44	0.01	0.00	0.00	0.05	0.00	0.00	0.04	2.2E-06	1.3E-06	2.5E-06	3.2E-06	2.9E-08
	Elderly (65–97 yr)	0.01	0.00	0.04	0.02	0.03	0.08	53.44	0.01	0.00	0.00	0.03	0.00	0.00	0.03	3.1E-06	1.9E-06	3.4E-06	4.5E-06	4.0E-08
**Cow milk**	Baby (0–2 yr)	0.34	0.19	12.18	**7.13**	8.70	1.44	1014.76	0.16	0.01	0.58	**10.19**	0.06	0.03	0.48	1.0E-06	2.5E-06	**1.2E-05**	**1.6E-05**	1.1E-08
	Children (3–9 yr)	0.06	0.04	2.28	1.33	1.63	0.27	189.60	0.03	0.00	0.11	**1.90**	0.01	0.01	0.09	1.2E-06	2.8E-06	**1.4E-05**	**1.8E-05**	1.3E-08
	Teenagers (10–17 yr)	0.02	0.01	0.82	0.48	0.59	0.10	68.58	0.01	0.00	0.04	0.69	0.00	0.00	0.03	9.9E-07	2.3E-06	**1.2E-05**	**1.5E-05**	1.1E-08
	Adult (18–64 yr)	0.01	0.01	0.36	0.21	0.26	0.04	29.80	0.00	0.00	0.02	0.30	0.00	0.00	0.01	1.3E-06	3.0E-06	**1.5E-05**	**2.0E-05**	1.4E-08
	Elderly (65–97 yr)	0.01	0.01	0.45	0.26	0.32	0.05	37.15	0.01	0.00	0.02	0.37	0.00	0.00	0.02	3.1E-06	7.3E-06	**3.7E-05**	**4.8E-05**	3.4E-08
**Seafood products**	Baby (0–2 yr)	1.80	0.02	0.24	**12.60**	0.13	1.18	81.09	0.86	0.00	0.01	**2.38**	0.00	0.06	0.04	5.5E-06	2.7E-07	2.5E-07	2.3E-07	2.1E-08
	Children (3–9 yr)	1.68	0.02	0.22	**11.77**	0.12	1.10	75.74	0.80	0.00	0.01	**2.23**	0.00	0.05	0.04	**3.1E-05**	1.5E-06	1.4E-06	1.3E-06	1.2E-07
	Teenagers (10–17 yr)	1.27	0.01	0.17	**8.86**	0.09	0.83	57.03	0.60	0.00	0.01	**1.68**	0.00	0.04	0.03	**5.4E-05**	2.7E-06	2.4E-06	2.35E-06	2.0E-07
	Adult (18–64 yr)	0.70	0.01	0.09	**4.92**	0.05	0.46	31.63	0.33	0.00	0.00	0.93	0.00	0.02	0.02	**8.8E-05**	4.4E-06	3.9E-06	3.8E-06	3.3E-07
	Elderly (65–97 yr)	0.53	0.01	0.07	**3.74**	0.04	0.35	24.06	0.25	0.00	0.00	0.71	0.00	0.02	0.01	**1.3E-04**	6.5E-06	5.9E-06	5.6E-06	5.0E-07
**Total**	Baby (0–2 yr)	2.50	0.25	13.22	**20.21**	9.40	2.27	1399.12	**1.19**	0.01	0.63	**13.25**	0.07	0.11	**1.05**					
	Children (3–9 yr)	1.91	0.07	2.91	**13.34**	2.03	1.47	547.16	0.91	0.00	0.14	**4.47**	0.02	0.07	0.39					
	Teenagers (10–17 yr)	1.40	0.04	1.27	**9.51**	0.87	1.04	293.89	0.67	0.00	0.06	**2.60**	0.01	0.05	0.24					
	Adult (18–64 yr)	0.78	0.02	0.63	**5.23**	0.43	0.59	178.66	0.37	0.00	0.03	**1.38**	0.00	0.03	0.15					
	Elderly (65–97 yr)	0.62	0.02	0.69	**4.10**	0.48	0.47	159.77	0.29	0.00	0.03	**1.23**	0.00	0.02	0.14					

*Significant values are indicated in bold*.

The CR_As_, CR_Cr_, and CR_Ni_ exceed the acceptable lifetime risk of 10^−5^ in beef, milk and seafood products. In particular, beef ingestion determined a certain risk for “elderly,” while cow milk consumption showed critical values of CR_Cr_ and CR_Ni_ for the all age classes. Furthermore, the CR_As_ related to seafood ingestion indicated a risk for all age classes, excepted for “baby” (Table 6).

The cow milk mainly contributed to the EWI of PAHs, determining a MOE value < 10,000 in the “baby” age class (7561; Table 7). The THQ and CR values due to PAHs ingestion did not show relevant risk for any foodstuff and age class.

**Table 7:** Estimated weekly intake (EWI; μg/kg b.w.), Target hazard quotient (THQ), Total Target hazard quotient (TTHQ), Target carcinogenic risk (CR) and Margin-of-exposure (MOE) for each PAH and their sum in the five age-categories.

**Food**		**EWI (ng/kg BW)**					**THQ**	**CR**	**MOE**
		**BaA**	**BaP**	**BbF**	**Chr**	**Σ4 PAH (BEC)**	**Σ4 PAH (BEC)**	**Σ4 PAH (BEC)**	**Σ4 PAH (BEC)**
**Beef**	Baby (0–2 yr)	2.20	2.20	2.20	7.39	2.71	0.00	5.5E-09	258157
	Children (3–9 yr)	0.73	0.73	0.73	2.45	0.90	0.00	1.1E-08	780009
	Teenagers (10–17 yr)	0.57	0.57	0.57	1.91	0.70	0.00	2.0E-08	1001493
	Adult (18–64 yr)	0.35	0.35	0.35	1.18	0.43	0.00	3.6E-08	1622419
	Elderly (65–97 yr)	0.38	0.38	0.38	1.27	0.47	0.00	7.7E-08	1502240
**Pork**	Baby (0–2 yr)	0.09	0.09	0.09	0.09	0.11	0.00	2.3E-10	6357279
	Children (3–9 yr)	0.43	0.43	0.43	0.43	0.53	0.00	6.4E-09	1331877
	Teenagers (10–17 yr)	0.22	0.22	0.22	0.22	0.27	0.00	7.8E-09	2580512
	Adult (18–64 yr)	0.17	0.17	0.17	0.17	0.20	0.00	1.7E-08	3440683
	Elderly (65–97 yr)	0.12	0.12	0.12	0.12	0.14	0.00	2.4E-08	4857434
**Cow milk**	Baby (0–2 yr)	105.80	34.80	34.80	4371.38	92.58	0.04	1.9E-07	**7561**
	Children (3–9 yr)	19.77	6.50	6.50	816.78	17.31	0.01	1.1E-07	40437
	Teenagers (10–17 yr)	7.15	2.35	2.35	295.41	6.26	0.00	1.8E-07	111803
	Adult (18–64 yr)	3.11	1.02	1.02	128.36	2.72	0.00	2.3E-07	257300
	Elderly (65–97 yr)	3.87	1.27	1.27	160.01	3.39	0.00	5.6E-07	206405
**Seafood**	Baby (0–2 yr)	5.10	5.10	5.10	5.10	6.17	0.00	1.3E-08	113523
	Children (3–9 yr)	4.76	4.76	4.76	4.76	5.76	0.00	7.0E-08	121536
	Teenagers (10–17 yr)	3.58	3.58	3.58	3.58	4.34	0.00	1.2E-07	161415
	Adult (18–64 yr)	1.99	1.99	1.99	1.99	2.41	0.00	2.0–07	291002
	Elderly (65–97 yr)	1.51	1.51	1.51	1.51	1.83	0.00	3.0E-07	382614
**Total**	Baby (0–2 yr)	113.19	42.19	42.19	4383.96	101.57	0.05		
	Children (3–9 yr)	25.69	12.43	12.43	824.42	24.49	0.01		
	Teenagers (10–17 yr)	11.53	6.73	6.73	301.13	11.57	0.01		
	Adult (18–64 yr)	5.61	3.53	3.53	131.70	5.76	0.00		
	Elderly (65–97 yr)	5.88	3.28	3.28	162.92	5.83	0.00		

*The total BaP equivalent concentration (BEC) and an exposure time of 365 days year^−1^ were considered. The Significant values are indicated in bold*.

## Discussion

The foodstuffs of animal origin (terrestrial and aquatic) play a key-role in the human diet as they provide proteins, vitamins, and other important nutrients (59, 100) with potential health benefits. Otherwise, their consumption represents the principal pathway of exposure to potentially deleterious compounds, such as heavy metals and persistent organic pollutants, constituting therefore an important public health issue (50, 59). Indeed, animals are constantly exposed to contaminants present in the environment and are able to accumulate pollutants in elevated concentration in their tissues, in particular in the fat (53, 101). Therefore, the EU Scientific Committee on Food has set maximum level for certain contaminants in foodstuffs, periodically monitoring by the European Food Safety Authority (EFSA).

In this preliminary study, we evaluated the content of heavy metals and PAHs in local products from a contaminated site (SNI of Augusta), in order to assess the risk for resident population derived from their consumption.

### Occurrence of Heavy Metals in the Study Area

This investigation shows that heavy metal concentrations were not relevant in meat and milks and that seafood products give the main contribution to the total dietary uptake.

Cd and Pb, concentrations were below the regulation limit in all matrices analyzed (1) and along with Cr, Ni, and Zn were generally consistent with those reported by EFSA (102–106). Differently, the pork meat showed a higher concentration of Pb (mean 0.024 μg/g) respect EFSA value [0.011 μg/g; (103)] due to the highest concentration (0.064 μg/g) found in one sample collected in a farm very close to the SNI center. Moreover, Cr and Ni concentrations were lower than those found in meats collected in Europe (104, 105). Data on contaminant concentrations in terrestrial foodstuffs were also compared with those collected (IZSSi database) during the National Residual Plan carried out in Sicily from 2012 to 2015. Ni and As contents in dairy products collected in the SNI area (municipalities of Siracusa) were lower than those found in the same food category in other districts of Sicily (*P* < 0.05; Table 8), while heavy metals in meats did not show significant difference.

**Table 8:** Significative difference by Mann-Whytney's test (*P*-value < 0.05).

**Metal**	**Samples from the SNI area *n***	**Samples from other districts *n***	**SNI area mean score**	**Other districts mean score**	***P*-value**
Arsenic (As)	18	7	9.50	22.00	<0.0001
Nickel (Ni)	18	6	9.88	20.33	0.0002

Hg concentrations here found in seafood products are of great concern, exceeding the limits set for fishery products by the European Regulation (1). The Hg content in meats and milks was < LOD, according to EFSA (73) that considers only Hg content in fish and other seafood for the evaluation of human exposure.

The higher Hg concentrations measured in seafood samples is probably related to ecological habits of species and are consistent with results reported by previous studies in the same area (23, 39, 43, 107). Specifically, Bonsignore et al. (43) have found elevated Hg concentrations in fish caught from inside and outside the Augusta Bay, suggesting an active release mechanism of Hg from sediments to the environment and thus a potential risk, both for marine organisms and consumer, due to seafood ingestion.

Arsenic, particularly in the inorganic form, is classified by IARC as carcinogenic to humans [Group 1, (108, 109)] and thus is often considered for human health risk assessment, although there is no legislation defining As limit in food.

Polluted water and soil are the principal pathways carrying As to the food chain. Thus, intake of As contaminated food is a growing issue of public concern (60, 89, 110–113). In this study As was detected in most of foodstuffs samples, with a variable range of concentration. The higher values were recorded in seafood products. These concentrations fall within the range found in seafood products in other Mediterranean areas (114–116), while the estimated iAs concentration (0.14 μg/g) was higher than value recorded by EFSA [0.025 μg/g; (89)] for seafood products, probably due to the specific environmental condition. Beef and pork showed an estimated iAs average contents (0.008 and 0.010 μg/g, respectively) similar to those found by EFSA (89) in livestock meats (mean 0.006 μg/g).

### Occurrence of PAHs in the Study Area

In this study, detectable Chr concentrations were found in all terrestrial foodstuffs samples, with higher concentrations detected in raw milks. These results were compared to the European limits (1, 117) that are set on the maximum levels for benzo(a)pyrene and on the sum of the four PAHs (benzo(a)pyrene, benz(a)anthracene, benzo(b)fluoranthene, and chrysene). This approach was introduced by European Community (117) to guaranty not entering in the market products in which BaP is not detectable, but where others PAH are present. Although there aren't PAHs limits referred specifically to raw milk, Chr showed higher values both in cow (mean 12.56 ng/g), sheep and goat milks (mean 9.25 ng/g) compared to the lowest maximum food concentrations set for infants and young children [1 ng/g; (117)]. Chr and BaA are classified as IARC group 2B, being considered an agent (mixture) possibly carcinogenic to humans. Chr concentrations here found in milk samples are similar to those found in cow milk collected in polluted Kuwait area (54) and in smoked dairy products (99), but much higher than those found in unprocessed and heat-treated cow milk in south Italy and Europe (17, 54, 118), breast milk and infant formula (18) and dairy products (58). Otherwise, BaA concentrations here found in milk samples (< LOD) are similar to those found in other investigations (54, 58) and lower than those found in milk from European industrial areas, where values until 1.5 ng/g were registered (54).

Since the food contamination by PAHs is related also to industrial food processing and some cooking treatment, the presence of PAHs in raw matrices nearest the SNI, reflects a certain level of environmental contamination.

The limits for PAHs concentrations in meats and seafood are set only for smoked products [2 ng/g and 12 ng/g for BaP and ∑4PAH, respectively; (117)]. Concentrations here found in unprocessed products were much lower than the maximum level set for smoked products and consistent with those observed in other studies carried out in Europe (54, 58, 99).

### Human Health Risk Assessment

The human health risk assessment based on the evaluation of EWI, THQ, and CR showed a certain degree of risk, principally related to Hg exposure. Particularly, the elevated values of the indices EWI_Hg_ (for all the age categories) and THQ_Hg_ (for baby, children, and teenagers) estimated for seafood category suggested that consumption of these products from the Augusta bay is not recommended, especially for more susceptible categories.

The CR_As_ related to seafood ingestion exceed the threshold limit (1 × 10^−5^) for all age categories, (except for the “baby”) indicating a risk for consumers. As previously mentioned, we estimated the iAs content applying a specific percent to the total As but, until now, it is not possible to certainly predict the inorganic content of iAs in seafood (89, 119). Further investigations are recommended to clarify this issue.

We used a conservative approach for human exposure considering a value equal to ½ LOD for contaminants with concentration < LOD. Therefore, the significant value obtained for EWI_Hg_, THQ_Hg_, CR_Ni_, and CR_Cr_ by cow milk and beef ingestion (Table 5) could be overestimated and need to be carefully considered.

Among the risk indices calculated for PAHs, the margin-of-exposure approach (MOE) was significant (7561) for “baby” by cow milk ingestion, probably related to the high Chr content found in this food and the elevated ingestion rate from this age category (Table 7).

## Conclusions

This study represents a first investigation of heavy metals and PAHs concentrations in different foodstuffs from the SNI of Augusta-Melilli-Priolo. Results indicated that the seafood exceeded the mercury limits established by the European legislation and contributed more respect the other foodstuffs to the heavy metals dietary intake. Otherwise, the terrestrial matrices, in particular milks, presented significant contents of chrysene reaching higher values than those set for food for infants and young children by European legislation. The high heavy metals concentration in seafood as well as the high PAHs concentrations in raw foodstuff sampled near the SNI suggest an environmental contamination of the Augusta Bay due to anthropogenic activities. The evaluation of human health risk related to seafood products consumption evidenced the overcoming of Provisional Tolerable Weekly Intake (PTWI) for Hg recommended by the European Food Safety Authority and WHO, and a non-carcinogenic risk (THQ) for Hg intakes occurs in baby, children and teenagers. The arsenic cancer risk (CR_As_) exceeded the threshold limit for almost age categories (except “baby”) and for elderly, due to seafood products and beef ingestions, respectively. Finally, the margin-of-exposure calculated for “baby” showed a certain cancer risk due to cow milk ingestion, probably related to the high chrysene content found in this food and the elevated ingestion rate from this age category.

The consumption of local animal foodstuffs, in particular seafood, should represent a risk for local population health, and further studies are recommended to evaluate the contaminants' exposure, especially for certain vulnerable categories of consumers.

## Data Availability Statement

The datasets presented in this article are not readily available because they are part of an ongoing project. Requests to access the datasets should be directed to www.cisas.cnr.it.

## Author Contributions

CD, AT, and CG made substantial contributions to conception and design of the study, as well as the monitoring plan. They drafted and critically revised the manuscript for its intellectual content, gave final approval of the version to be published and agreed to be accountable for all aspects of the work. VF, DC, GL, AB, MD, FF, SG, and MU made substantial contributions to foodstuffs analysis and data acquisition. Each of the authors read and approved the final version of the manuscript. All authors contributed to the article and approved the submitted version.

## Conflict of Interest

The authors declare that the research was conducted in the absence of any commercial or financial relationships that could be construed as a potential conflict of interest.
